# The Dynamics of Paid and Unpaid Activities Among People Aged 50–69 in Denmark, France, Italy, and England

**DOI:** 10.1177/0164027516654521

**Published:** 2016-06-14

**Authors:** Giorgio Di Gessa, Emily Grundy

**Affiliations:** 1 Department of Social Science, Health and Medicine, Institute of Gerontology, King’s College London, London, UK; 2 Department of Social Policy, The London School of Economics and Political Science, London, UK

**Keywords:** ELSA, SHARE, dynamics of engagement, paid work, formal activities, informal activities

## Abstract

In the context of the current policy emphasis on extending working lives, we investigate whether the relationship between participation in paid work, other formal, and informal activities among people aged 50–69 is complementary or competitive. We also investigate differences in associations between countries using comparable longitudinal data from Denmark, France, Italy, and England. We find positive associations between informal and formal engagement in cross-sectional and longitudinal analyses. Paid work was negatively associated with formal and informal engagement, and respondents who stopped working were more likely to be engaged in formal (Denmark and France) and informal activities (England and Italy) at follow-up than respondents who continued working. However, the strongest predictor of formal and informal engagement at follow-up was baseline engagement. In the context of policy aims to extend working lives and broaden older people’s participation in other productive activities, new balances between work and other forms of engagement are still to be found.

Population aging—a major demographic trend throughout Europe—has implications for the organization and funding of public services predominantly used by older people and also for support ratios which in turn influence government revenues from taxation (European Commission, 2006). In response, many governments are pursuing policies designed to promote employment and extend working lives (Vickerstaff, 2010). In particular, many European countries are introducing reforms aimed at postponing ages of eligibility for state pensions. In the UK, for example, the state pension age for women has risen from age 60 for those born in 1950 or earlier to 66 for those born in 1955 and is set to increase further for both women and men (Ginn, 2013).

There is, however, an increasing recognition that the participation of older people in activities beyond the sphere of formal employment is important (http://ec.europa.eu/archives/ey2012/). Participation in a range of activities such as informal caregiving and volunteering may benefit society as a whole and older individuals themselves, offering meaningful, valued, and rewarding roles which enhance well-being (Walker & Maltby, 2012; World Health Organization [WHO], 2002). Additionally, older people’s engagement in clubs and organizations may be regarded as a contribution to the enhancement and development of social and cultural capital which benefits society as a whole (Putnam, 1995; Stolle & Rochon, 1998). However, opportunities for participation in such activities may be constrained by the current policy emphasis on extending working lives if the demands of work limit the time and motivation of older people to take up other activities (Chambré, 1984).

In this article, we investigate the interplay between paid work, formal, and informal engagement using data from four nationally representative European longitudinal studies. In particular, we examine whether participation in paid work is associated with lower levels of engagement in activities outside the labor market, and how changes in employment status are associated with changes in other forms of activity. To date, there is little literature which considers the interrelationship between paid work, formal, and informal activities and the way they may impact on one other. Moreover, the existing literature is largely based on cross-sectional data and does not consider the influence of varying contexts.

## Background

Previous cross-sectional studies suggest complex relationships between various forms of engagement at older ages. Vlachantoni (2010), using the third wave of the English Longitudinal Study of Aging (ELSA), found that men and women aged 50–64 who were economically active were less likely to provide care compared to those outside the labor force. Similarly, several studies using baseline data from the Survey of Health, Aging, and Retirement in Europe (SHARE) have reported that older people in paid work are less likely to provide informal help or care or to volunteer (Di Gessa, Glaser, Price, Ribe, & Tinker, 2015; Erlinghagen & Hank, 2006; Hank & Buber, 2009; Hank & Stuck, 2008). However, other studies mainly from North America have reported either no significant associations between paid work and volunteering or showed that those in paid work were more likely to engage in volunteering compared to their nonemployed counterparts (Caro & Bass, 1997; Chambré, 1984; Choi, 2003; Fischer, Mueller, & Cooper, 1991).

Longitudinal studies yield similarly mixed results; they tend to show a positive association between changes in employment status and formal volunteering, but no clear association with provision of informal help, suggesting that older people help friends and relatives regardless of their employment status. Several studies from North America have suggested that older people are more likely to take up formal activities such as volunteering if they were nonworkers or had stopped work than if they were full-time workers (Caro & Bass, 1997; Moen, Erickson, Argarwal, Fields, & Todd, 2000; Mutchler, Burr, & Caro, 2003). Similarly, using longitudinal data from SHARE, both Kohli, Hank, and Kunemund (2009) and Hank and Erlinghagen (2010) found that the probability of starting active participation in voluntary associations, educational courses, sport, social, or other kinds of clubs was highest in the immediate postretirement period, that is, among those who left the paid labor force between waves of the survey. However, retirement was not associated with uptake of informal activities such as providing help to friends and family when they retired.

Although previous longitudinal analyses based on SHARE seem to support the idea that older people in Europe who have left paid work are more likely to take up formal activities rather than informal ones, these studies have pooled results, assuming that the direction of the association between variables remains the same within each country (Allerbeck, 1977), which may not be the case. Erlinghagen (2010), for example, focused on a single country using the German Socio-Economic Panel and found that changes over time in employment status were *not* associated with the propensity to take up or end formal voluntary work. The author noted that the findings might not apply to other countries, given that older people’s participation in volunteering might be shaped by country-specific arrangements and policies.

Engagement of older people occurs within a sociopolitical context and may be influenced by social and economic policies and the provision of services, as well as norms and values. Policies related to work and retirement incentives and pension ages are clearly likely to influence participation in the labor market (Organisation for Economic Co-operation and Development [OECD], 2013). Similarly, the level and type of state provided services, including welfare benefits, elder care, and child care may influence participation in other types of activity including volunteering (Salamon & Sokolowski, 2001; Warburton & Jeppsson Grassman, 2011) and caregiving (Di Gessa et al., 2015; Hank & Buber, 2009; Hank & Erlinghagen, 2010; Lewis, Campbell, & Huerta, 2008). Engagement in activities such as volunteering tends to be higher where social expenditure and civic culture are high and where infrastructures and opportunities are available for people to be engaged in formal activities (Hank, 2010; Leonard & Johansson, 2008; Warburton & Jeppsson Grassman, 2011). Furthermore, there is evidence that in countries where the provision of informal and family care is high, participation in activities such as volunteering is reduced, as it is hypothesized that in such contexts social capital is concentrated in the family (Kohli, Hank, & Kunemund, 2009; Pichler & Wallace, 2007).

In this article, we analyze associations between three types of engagement (paid work, formal, and informal activities) in four European countries. The specific research questions we address are first whether or not the contemporaneous association between participation in different activities is positive or negative. The second related question is whether stopping paid work is associated with increased participation in other activities. Our final question is whether or not there are variations between the four countries under consideration in the direction and strength of cross-sectional and longitudinal associations suggestive of contextual influences on associations. Based on previous studies, we hypothesize that respondents in paid work would be less likely to engage in other activities and that those who stop working would be more likely to participate in other formal or informal activities. In terms of country differences, we expect that uptake of informal activities would be more likely in Southern European countries (in this case Italy), where social capital is concentrated in the family.

## Study Populations

Family systems and welfare state regimes are two dimensions which may influence older people’s patterns and levels of engagement. It is recognized that efforts at categorizing European countries along these dimensions represent something of a stylized oversimplification and that categorizations vary (Arts & Gelissen, 2002; Powell & Barrientos, 2004; Reher, 1998). However, most typologies distinguish social democratic countries with generous welfare provisions (such as Denmark and Sweden), Bismarckian countries (such as France, Germany, and Austria), countries with liberal and mixed structures (such as the UK, Ireland, and the United States), and a Mediterranean group (such as Spain, Italy, and Greece) in which welfare policies are premised on greater familial exchange (Arts & Gelissen, 2002; Powell & Barrientos, 2004). Lower levels of familialism—defined by geographic proximity, support, resource transfers, and obligations among family members—are generally observed in the Nordic, Western–Central, and British–Irish countries compared to Mediterranean countries, where family bonds and support are stronger (Dykstra & Fokkema, 2011; Pichler & Wallace, 2007; Reher, 1998).

In this article, we compare Denmark, France, Italy, and England, selected to “represent” different institutional, sociopolitical, and cultural contexts. Although we acknowledge that none of these countries typifies an ideal welfare state regime or family system, there are clear differences between them in retirement and labor market policies, for example (Bambra & Eikemo, 2009; Powell & Barrientos, 2004). In Denmark, in 2004, the official age of retirement was 65 for both men and women, whereas in Italy women were entitled to retire at 60 (compared to 65 for men). In Denmark and the UK, the effective and statutory ages of retirement were remarkably close (at about 64, compared to the official retirement age of 65), whereas in Italy men left the workforce on average at age 61 despite an official retirement age of 65 (OECD, 2006). Pragmatic consideration of data quality (detailed below) also prompted selection of these countries.

## Data and Methods

The data used were drawn from SHARE and ELSA; these are nationally representative longitudinal studies of the population aged 50 and over which by design have similar structures and content. Details on methodology, weighting strategies, and questionnaires have been reported elsewhere (Börsch-Supan & Jürges, 2005; Taylor et al., 2007). We selected for inclusion in the study Denmark, France, Italy, and England not only because these countries typify different institutional, sociopolitical, and cultural contexts but also because—compared with the other available countries from the same contextual regions—these countries had both good initial response rates (81% in France, 63% in Denmark, and 55% in Italy) and retention rates (63% in France, 73% in Denmark, and 69% in Italy). For instance, Germany had an initial response rate of 63% with an attrition rate of almost 50%; similarly, Belgium and Sweden with relatively high retention rates (73% and 66%, respectively) had very low initial response rates (39% and 47%, respectively).

We decided to use data from the first two waves of data collection in England, Denmark, France, and Italy, as data quality checks have shown that baseline data are broadly representative of national populations (Börsch-Supan & Jürges, 2005; Steptoe, Breeze, Banks, & Nazroo, 2012), whereas later rounds represent survivor samples depleted by attrition. ELSA collected Wave 1 information in 2002/2003, whereas SHARE collected Wave 1 data in 2004/2005; for both studies, later waves were conducted biennially. The analysis is based on respondents aged 50–69 at baseline who also participated in Wave 2 of the relevant study. This age-group was selected, as only very small proportions of those in age-groups older than this were in paid employment, and because withdrawal from the paid labor force most commonly occurs in this age range (OECD, 2013). Combining both data sets together, a total of 12,440 individuals were successfully interviewed at baseline, with 9,575 (76.9%) reinterviewed at Wave 2. Among the age-eligible participants interviewed in Wave 1, 201 (1.6%) died between waves and 21.5% declined to be interviewed at follow-up or could not then be traced. Consistent with other studies of survey attrition (Banks, Muriel, & Smith, 2011; Fitzgerald, Gottschalk, & Moffit, 1998), those who dropped out of the study before the second wave were generally more likely to be in poor health, with low education and in the poorest wealth quintile, and less likely to be engaged in formal activities at baseline.

### Measures of Engagement

Three major areas of activity were included in our definition of engagement: *paid work, formal engagement*, and *informal engagement.* In both surveys, respondents were classified as in paid work if they described their current situation as “employed or self-employed (including working for a family business)” and if they were not “temporarily away from any work, including seasonal work.” For those in paid work, we distinguished between those working full time (30 or more hours per week) and part time (less than 30 hr per week). Formal engagement was conceptualized to include nonkin social activities linked to formalized associations or groups, performed within an established structure with a regular schedule. Formal engagement as defined here thus included voluntary work; attendance at training courses; and participation in political organizations, religious organizations or sport, social, or other kinds of clubs. Informal engagement included activities with family members and/or friends, such as care provision for sick or disabled adults, provision of help to family, friends or neighbors, and looking after grandchildren without the presence of their parents. Given the small number of respondents who attended educational training courses or took part in political organizations, it was not possible to consider each activity separately. However, if we selected only the most frequent activities, we would not capture the contribution that older people make to society by maintaining an active role in a wider range of social, cultural, and civic affair activities. In our analyses, we therefore decided to include *any* participation in the activities recorded in the data. SHARE asked whether respondents participated in these activities monthly, almost every week, almost daily, or less often. ELSA included questions about fewer activities than SHARE and in less detail (for instance, there were only questions on membership of political, religious, or social organizations, not on frequency of attendance and participation) and also used a different temporal reference period, in most cases asking about activities in the month prior to interview. As a result in ELSA, formal engagement included only volunteering and attendance at formal educational or training course (in the past month), and informal engagement included caring for a sick or disabled adult (in the past month) and looking after anyone (in the past week). Although at some cost in terms of comparability with ELSA, we categorized SHARE respondents as engaged in the relevant activity if they reported participation almost weekly or daily, as such indicators of more intensive engagement were considered more useful in addressing our specific research questions.

In order to examine changes in participation in formal and informal activities between waves of the survey, we distinguished between those engaged in the activity at both waves, those who dropped the activity (engaged at Wave 1 but not at Wave 2), those who took up the activity (not engaged at Wave 1 but engaged at Wave 2), and those not engaged at either time point. The classification derived to look at changes in paid work was slightly different as only 3% (*N* = 308 across all four countries) of those not in paid work at Wave 1 were working at Wave 2. This group was too small to distinguish separately in the analysis, we therefore derived a variable distinguishing (1) those in paid work at Wave 2 (nearly all of whom had also been in paid work at Wave 1), (2) those in paid work at Wave 1 but not at Wave 2, and (3) those not in paid work at either wave. Because of the small numbers involved, it was not possible to capture transitions between part-time and full-time work.

### Covariates

Based on previous studies, demographic, socioeconomic, and health characteristics included in the analyses were gender, age, marital status, level of education, household wealth, self-rated health, and self-reported functional limitation. Participation in paid work tends not to decrease linearly with age, so we used a categorical indicator of age-group (50–54, 55–59, 60–64, 65–69). Marital status was measured using a dichotomized indicator of whether the respondent was married/cohabiting or not. Educational qualifications were grouped into three categories: low, mid, and high education using the International Standard Classification of Education (http://www.uis.unesco.org/), where a high level of education refers to university education or above. Wealth was measured using quintiles, for each country, derived by the RAND Corporation (www.mmicdata.rand.org/meta/) and based on the sum of the net value of properties, nonhousing financial wealth, and business assets. Information on self-rated health (SRH) was dichotomized into “fair” or “poor” versus “excellent,” “very good,” or “good.” Functional health was measured using a dichotomized variable indicating whether or not respondents had any limitations in activities of daily living (ADL).

### Statistical Analyses

Initially, we restricted analyses to participants with complete data on all variables examined. However, such an approach may lead to biased estimates, especially when attrition is higher than 10% as in our study, because complete case analysis involves the assumption that data are missing completely at random (MCAR) and that missingness is not related to observed or unobserved measurements (Fitzgerald et al., 1998; Marshall, Altman, Royston, & Holder, 2010). When missingness is related to some characteristics of the study sample, an appropriate way of addressing missing data issues is to use the multiple imputation (MI) approach which assumes that data are missing at random (MAR), rather than MCAR (Little & Rubin, 2002). This implies that all systematic selection effects depend on variables which are included in the model (such as baseline socioeconomic characteristics and health status which, as already noted, were strongly associated with attrition). Given the dichotomous nature of the outcome variables, we used logistic regression models. All analyses were performed using Stata, version 13, and adjust for survey design effects.

## Results

### Descriptive Statistics

Table 1 provides a description of the baseline samples and changes in participation in paid work, formal, and informal activities between waves. Educational level and health differed across samples; for instance, three quarters of the Italian respondents were in the lowest educational category as opposed to less than one fifth in Denmark. There was considerable variation in the level of engagement in paid work and formal activities across the four countries. For instance, 54% of respondents in Denmark were in paid work compared to 47% of those in England, 43% of the French sample, and 25% of respondents in Italy. The proportion engaged in formal activities was also much higher in Denmark than in France or Italy. Proportions engaged in informal activities were similar in Denmark, France, and Italy but lower in England. However, as mentioned above, ELSA respondents were not asked comparable questions. With respect to changes in participation between waves, only about 15% of respondents had taken up engagement in formal or informal activities by follow-up, whereas in all countries roughly 10% of the sample left paid work between waves.

**Table 1.** Distribution of the Sample by Baseline Characteristics and Changes in Participation Between Waves (%), by Country.

	Denmark	France	Italy	England
Baseline Characteristics				
In paid work full time	44.6	34.6	18.4	32.9
In paid work part time	9.5	8.9	7.0	14.0
Formally engaged	37.3	26.1	12.7	17.1
Informally engaged	40.3	39.5	40.8	23.5
Not engaged in any activity	17.3	24.9	37.3	32.4
Engaged in all activities	6.8	4.6	1.5	2.0
Female (%)	50.5	52.5	56.3	53.4
Age (mean)	58.4	58.5	60.0	59.1
Education: low	17.9	43.3	72.8	39.4
Education: middle	46.9	34.3	18.4	34.6
Education: high	35.2	22.4	8.8	26.0
Married (%)	69.0	72.4	83.0	73.7
Self-rated health poor or fair (%)	21.4	25.4	33.5	23.4
Activities of daily living limitations (%)	7.0	6.3	5.7	15.5
No. of observations at baseline	1,102	1,999	1,776	7,563
Changes in Participation between Waves 1 and 2				
In paid work at both waves	45.2	34.8	18.0	40.9
Stopped paid work by Wave 2	12.6	9.1	8.1	9.8
No paid work at either wave	42.2	55.9	73.9	49.3
Stopped formal engagement by Wave 2	10.2	11.1	5.3	8.3
No formal engagement at either wave	39.7	59.6	77.3	71.5
Started formal engagement by Wave 2	20.7	12.1	8.9	10.1
Formally engaged at both waves	29.4	17.2	8.4	10.1
Stopped Informal engagement by Wave 2	18.1	17.0	12.8	14.7
No informal engagement at either wave	42.3	44.0	41.7	68.8
Started informal engagement by Wave 2	17.0	14.2	16.0	7.8
Informally engaged at both waves	22.6	24.8	29.5	8.7
No. of observations to both waves	866	1,369	1,291	6,049

*Source*. Denmark, France, and Italy data obtained from Survey of Health, Aging, and Retirement in Europe (2004, 2006); English data obtained from English Longitudinal Study of Aging (2002, 2004). Study samples for changes in participation are restricted to those who responded to both interviews.

*Note*. Measures of formal and informal engagement relate to past week in Denmark, France, and Italy and past month in England. Own calculations.

### Cross-Sectional Multivariate Findings

The relationships between the three forms of activities at baseline were investigated using multivariate logistic regression. Tables 2 and 3 show results from the fully adjusted logistic regression model of participation at baseline in formal and informal activities, respectively, controlling for engagement in the other two forms of activity as well as for the sociodemographic and health indicators.

**Table 2.** Participation in Formal Activities at Baseline, Controlling for Baseline Socioeconomic and Demographic Characteristics and for Engagement Variables.

Variable	Denmark		France		Italy		England	
	*OR*	95% CIs	Or	95% cis	*OR*	95% CIs	*OR*	95% CIs
Female^a^	0.92	[0.70, 1.21]	0.80	[0.64, 1.01]	0.84	[0.60, 1.16]	1.55***	[1.35, 1.78]
Age: 55–59^b^	1.00	[0.71, 1.42]	1.10	[0.80, 1.47]	0.81	[0.50, 1.31]	1.02	[0.86, 1.21]
Age: 60–64^b^	1.03	[0.70, 1.52]	1.17	[0.82, 1.68]	1.06	[0.64, 1.74]	0.98	[0.81, 1.20]
Age: 65–69^b^	1.04	[0.66, 1.65]	1.04	[0.70, 1.56]	0.82	[0.48, 1.40]	1.02	[0.83, 1.26]
Education: middle^c^	1.09	[0.75, 1.60]	1.52***	[1.17, 1.98]	2.47***	[1.70, 3.60]	2.80***	[2.35, 3.34]
Education: high^c^	1.93***	[1.30, 2.86	2.97***	[2.22, 3.98]	2.54***	[1.55, 4.18]	5.71***	[4.76, 6.85]
In poorest quintile^d^	0.71	[0.49, 1.05]	0.66**	[0.48, 0.94]	0.83	[0.52, 1.29]	0.75**	[0.60, 0.94]
Married^e^	1.13	[0.84, 1.52]	1.01	[0.78, 1.30]	0.66**	[0.45, 0.97]	1.04	[0.89, 1.21]
SRH poor or fair^f^	0.73	[0.51, 1.06]	0.65***	[0.48, 0.86]	0.76	[0.52, 1.10]	0.67***	[0.56, 0.81]
ADL limitations^g^	0.65	[0.35, 1.17]	0.85	[0.50, 1.44]	0.94	[0.43, 2.05]	0.89	[0.71, 1.10]
In paid work part time^h^	0.57**	[0.33, 0.96]	0.52***	[0.32, 0.83]	1.14	[0.65, 2.02]	0.73***	[0.59, 0.89]
In paid work full time^h^	0.63***	[0.44, 0.89]	0.68***	[0.50, 0.92]	0.52***	[0.31, 0.87]	0.63***	[0.53, 0.75]
Informally engaged^i^	1.43***	[1.09, 1.85]	1.73***	[1.38, 2.15]	1.77***	[1.29, 2.42]	1.39***	[1.21, 1.61]
Constant	0.54**	[0.30, 0.96]	0.26***	[0.17, 0.42]	0.17***	[0.08, 0.31]	0.07***	[0.06, 0.10]
No. of observations	1,102		1,999		1,776		7,563	

*Source*. Survey of Health, Aging, and Retirement in Europe (2004) and English Longitudinal Study of Aging (2002).

*Note*. Measures of engagement relate to past week in Denmark, France, and Italy and past month in England. Own calculations. Odds ratios (*OR*s) and 95% confidence intervals (CIs) obtained from fully adjusted logistic regression, by country.

^a^Male. ^b^50–54. ^c^Low education. ^d^In other wealth quintiles at baseline. ^e^Not married at baseline. ^f^Self-rated health (SRH) at baseline = good, very good, or excellent. ^g^No activities of daily living (ADL) limitations at baseline. ^h^Not in paid work at baseline. ^i^Not informally engaged at baseline.

** and ***Significant at the .05 and .01 levels, respectively.

**Table 3.** Participation in Informal Activities at Baseline, Controlling for Baseline Socioeconomic and Demographic Characteristics and for Engagement Variables.

Variable	Denmark		France		Italy		England	
	*OR*	95% CIs	*OR*	95% CIs	*OR*	95% CIs	*OR*	95% CIs
Female^a^	1.24	[0.97, 1.64]	1.65***	[1.35, 2.02]	1.86***	[1.49, 2.42]	1.66***	[1.47, 1.88]
Age: 55–59^b^	0.81	[0.58, 1.13]	1.13	[0.87, 1.46]	1.01	[0.72, 1.41]	0.97	[0.84, 1.13]
Age: 60–64^b^	0.85	[0.59, 1.25]	1.02	[0.75, 1.40]	1.10	[0.77, 1.56]	0.80**	[0.67, 0.95]
Age: 65–69^b^	0.73	[0.46, 1.15]	1.05	[0.75, 1.47]	0.96	[0.67, 1.38]	0.72***	[0.60, 0.87]
Education: middle^c^	1.04	[0.72, 1.49]	1.18	[0.94, 1.47]	0.99	[0.73, 1.36]	1.02	[0.90, 1.17]
Education: high^c^	1.16	[0.79, 1.70]	0.97	[0.74, 1.27]	1.02	[0.68, 1.55]	0.89	[0.76, 1.04]
In poorest quintile^d^	0.98	[0.69, 1.40]	1.08	[0.83, 1.41]	1.05	[0.78, 1.36]	1.28***	[1.09, 1.50]
Married^e^	0.98	[0.74, 1.30]	1.19	[0.95, 1.49]	1.44**	[1.09, 1.87]	1.46***	[1.27, 1.67]
SRH poor or fair^f^	1.00	[0.70, 1.42]	0.89	[0.71, 1.14]	0.90	[0.71, 1.13]	0.91	[0.78, 1.06]
ADL limitations^g^	0.53**	[0.30, 0.94]	1.04	[0.69, 1.58]	0.86	[0.53, 1.35]	0.82**	[0.69, 0.97]
In paid work part time^h^	0.73	[0.44, 1.23]	0.84	[0.57, 1.23]	0.87	[0.56, 1.36]	0.75***	[0.63, 0.88]
In paid work full time^h^	0.66**	[0.47, 0.93]	0.72**	[0.58, 0.98]	0.59***	[0.42, 0.83]	0.52***	[0.44, 0.61]
Formally engaged^i^	1.42**	[1.09, 1.86]	1.73***	[1.38, 2.16]	1.75***	[1.28, 2.39]	1.39***	[1.20, 1.61]
Constant	0.73	[0.41, 1.27]	0.38***	[0.26, 0.57]	0.37***	[0.23, 0.59]	0.24***	[0.19, 0.31]
No. of observations	1,102		1,999		1,776		7,563	

*Source*. Survey of Health, Aging, and Retirement in Europe, 2004 and English Longitudinal Study of Aging, 2002.

*Note*. Measures of engagement relate to past week in Denmark, France, and Italy and past month in England. Own calculations. Odds ratios (*OR*s) and 95% confidence intervals (CIs) obtained from fully adjusted logistic regression, by country.

^a^Male. ^b^50–54. ^c^Low education. ^d^In other wealth quintiles at baseline. ^e^Not married at baseline. ^f^Self-rated health (SRH) at baseline = good, very good, or excellent. ^g^No activities of daily living (ADL) limitations at baseline. ^h^Not in paid work at baseline. ^i^Not informally engaged at baseline.

** and ***Significant at the .05 and .01 levels, respectively.

#### Participation in formal activities at baseline

Table 2 shows a significant positive association between formal and informal engagement in all the countries under study: Respondents engaged in informal activities at baseline were between about 1.40 (Denmark and England) and 1.77 (Italy) times more likely to participate in formal activities than those not informally engaged. However, engagement in formal activities was negatively associated with participation in both part-time and full-time paid work: Being in full-time paid work decreased the odds of participating in formal activities by a factor of between 0.52 (Italy) and 0.68 (France). Part-time work was also significantly associated with reduced participation in formal activities in all countries except Italy. With respect to the other demographic and socioeconomic variables, some common patterns were observed in all countries under study: No significant association was found between age and formal engagement; and higher education was positively associated with participation in formal activities. Respondents who reported their health as poor or fair, and those in the lowest wealth quintile were about 0.70 times less likely to report formal participation, although this association only met conventional levels of statistical significance in France and England. Women were significantly more likely to be formally engaged than men only in England (odds ratio [*OR*] = 1.55).

#### Participation in informal activities at baseline

Table 3 presents the results of the logistic regression model for participation in informal and family activities. This also shows a significant negative relationship between informal engagement and full-time paid work in all countries under study: The odds of being informally engaged for respondents in full-time paid work were between 28% (France) and 48% (England) lower than among those not in paid work. As for the association between part-time work and informal engagement, we found no significant association in the SHARE countries, but respondents in England who worked part-time were 0.75 times less likely to report informal engagement relative to respondents not in paid work. As for the demographic and socioeconomic characteristics, being female and being married were associated with higher odds of informal participation, although these associations were not significant in Denmark. Age, wealth, and education were not significantly associated with informal engagement in any of the SHARE countries. However, compared to those aged 50–54, ELSA respondents aged 60–64 or 65–69 (*OR*s = 0.74 and 0.83, respectively) were significantly less likely to be engaged in informal activities; ELSA respondents in the lowest wealth quintile were 1.27 times more likely to be informally engaged. Finally, Danish and English respondents with functional limitations were less likely to participate in informal activities (*OR*s = 0.53 and 0.82, respectively) compared to those who reported no ADL limitations.

### Longitudinal Multivariate Findings

Longitudinal relationships between formal (informal) engagement at follow-up and changes in engagement in paid work and informal (formal) activities between waves were investigated using multivariate logistic regression, controlling for baseline participation as well as baseline socioeconomic and demographic characteristics.

#### Participation in formal activities at follow-up

Table 4 shows a positive association between engagement in informal and formal activities. In all countries except Italy, respondents who stopped being informally engaged between waves and those not informally engaged at either wave were between 0.53 (Denmark) and 0.72 (England) times less likely to be formally engaged at follow-up than respondents engaged in informal activities at both waves. With regard to changes in paid work, in Denmark and France respondents who were no longer in paid work at follow-up increased the odds of being formally engaged at Wave 2 by a factor of 1.60 compared to those who were in paid work at both waves. In all countries, baseline participation in formal activities was the strongest predictor of formal engagement at follow-up, underscoring the continuity of formal engagement in later life. Including interaction terms between work status and formal engagement at baseline (results not shown) showed that work status had no substantial effects on formal engagement at follow-up among respondents who were already engaged at baseline. In all countries, respondents with university education and above were between 1.83 (France) and 3.03 (England) times more likely to be formally engaged than those with low levels of education.

**Table 4.** Participation in Formal Activities at Wave 2, by Baseline Socioeconomic and Demographic Characteristics and Changes in Engagement Variables.

Variable	Denmark		France		Italy		England	
	*OR*	95% CIs	*OR*	95% CIs	*OR*	95% CIs	*OR*	95% CIs
Female^a^	1.39**	[1.02, 1.88]	1.16	[0.89, 1.52]	1.06	[0.72, 1.57]	1.29***	[1.11, 1.50]
Age: 55–59^b^	1.32	[0.88, 1.97]	1.08	[0.77, 1.52]	1.26	[0.74, 2.16]	0.93	[0.77, 1.13]
Age: 60–64^b^	1.66**	[1.06, 2.61]	1.01	[0.67, 1.50]	1.46	[0.84, 2.54]	0.99	[0.79, 1.23]
Age: 65–69^b^	1.57	[0.92, 2.66]	1.14	[0.71, 1.83]	1.19	[0.66, 2.16]	1.16	[0.92, 1.47]
Education: middle^c^	1.36	[0.88, 2.11]	1.26	[0.91, 1.73]	1.68**	[1.08, 2.63]	1.80***	[1.49, 2.17]
Education: high^c^	1.95***	[1.23, 3.07]	1.83***	[1.28, 2.60]	2.52***	[1.42, 4.48]	3.03***	[2.48, 3.68]
In poorest quintile^d^	0.85	[0.54, 1.32]	0.76	[0.52, 1.11]	1.07	[0.68, 1.69]	1.06	[0.84, 1.34]
Married^e^	0.88	[0.63, 1.22]	1.04	[0.77, 1.41]	0.98	[0.60, 1.58]	1.17	[0.98, 1.38]
SRH as poor or fair^f^	0.68	[0.45, 1.02]	0.72	[0.56, 1.06]	0.74	[0.48, 1.09]	0.82	[0.68, 1.01]
ADL limitations^g^	0.47**	[0.23, 0.96]	0.99	[0.56, 1.74]	0.56	[0.23, 1.37]	0.79	[0.63, 1.01]
Formally Engaged at Wave 1^h^	5.81***	[1.24, 7.97]	7.29***	[5.59, 9.51]	10.9***	[8.55, 19.5]	6.82***	[5.85, 7.94]
Informally engaged at both waves	1.00	—	1.00	—	1.00	—	1.00	—
Stopped informal by Wave 2	0.53***	[0.33, 0.85]	0.68**	[0.46, 0.99]	1.02	[0.57, 1.88]	0.60***	[0.45, 0.81]
Started informal by Wave 2	0.97	[0.59, 1.58]	0.97	[0.61, 1.53]	1.06	[0.66, 1.71]	1.33	[0.98, 1.82]
No informal at either wave	0.60***	[0.41, 0.89]	0.69**	[0.48, 0.98]	0.72	[0.50, 1.04]	0.72***	[0.57, 0.91]
In paid work at both waves	1.00	—	1.00	—	1.00	—	1.00	—
Stopped paid work by Wave 2	1.57**	[1.02, 2.51]	1.61**	[1.00, 2.73]	1.33	[0.63, 2.78]	1.18	[0.91, 1.54]
No paid work at either wave	1.17	[0.72, 1.89]	1.29	[0.85, 1.97]	1.54	[0.96, 2.67]	1.12	[0.93, 1.36]
Constant	0.33**	[0.17, 0.63]	0.17***	[0.09, 0.30]	0.08***	[0.03, 0.16]	0.08***	[0.05, 0.11]
No. of observations	1,086		1,916		1,758		7,521	

*Source*. Survey of Health, Aging, and Retirement in Europe (2004, 2006); English Longitudinal Study of Aging (2002, 2004). Estimates are based on 20 imputed data sets under missing at random.

*Note*. Study samples are restricted to those who responded to both interviews. Measures of engagement relate to past week in Denmark, France, and Italy and past month in England. Own calculations. Odds ratios (*OR*s) and 95% confidence intervals (CIs) obtained from fully adjusted logistic regression, by COUNTRY.

^a^Male. ^b^50–54. ^c^Low education. ^d^In other wealth quintiles at baseline. ^e^Not married at baseline. ^f^Self-rated health (SRH) at baseline = good, very good, or excellent. ^g^No activities of daily living (ADL) limitations at baseline. ^h^Not formally engaged at baseline.

** and ***Significant at the .05 and .01 levels, respectively.

#### Participation in informal activities at follow-up

When informal participation was considered (Table 5), similar results were found. Respondents who stopped participation in formal activities between waves (in Denmark, France, and England) or who were not formally engaged at both waves (in all countries) were about 0.6 times significantly less likely to be informally engaged at follow-up than those who were engaged in formal activities in both waves. With regard to changes in paid work, in Italy and England respondents who stopped work between waves were significantly (*OR*s 1.54 and 1.38, respectively) more likely to be engaged in informal activities at Wave 2 compared to those who were in paid work at both waves. In addition, ELSA respondents who were not in paid work at either wave had higher odds of being informally engaged at Wave 2 (*OR* = 1.37). Finally, informal engagement at baseline was the strongest predictor of participation in informal activities at follow-up in all countries under study: Respondents who were informally engaged at baseline were between 3.41 (Denmark) and 5.14 (Italy) times more likely to be so at follow-up, again underlying the continuity in engagement in later life. In all countries, women were more likely to be informally engaged at Wave 2 than men, and there was a negative association between poor health and informal engagement in Italy and England, with the odds of informal engagement at follow-up for those in poor or fair health about 25% lower than for those in better health at baseline.

**Table 5.** Participation in Informal Activities at Wave 2, by Baseline Socioeconomic and Demographic Characteristics and Changes in Engagement Variables.

Variable	Denmark		France		Italy		England	
	*OR*	95% CIs	*OR*	95% CIs	*OR*	95% CIs	*OR*	95% CIs
Female^a^	1.41**	[1.05, 1.89]	1.43**	[1.13, 1.81]	1.65**	[1.28, 2.11]	1.78***	[1.53, 2.08]
Age: 55–59^b^	1.02	[0.69, 1.48]	1.11	[0.82, 1.52]	1.17	[0.82, 1.69]	0.81**	[0.67, 0.98]
Age: 60–64^b^	0.91	[0.58, 1.40]	1.41	[0.96, 2.06]	1.23	[0.84, 1.80]	0.83	[0.66, 1.03]
Age: 65–69^b^	0.83	[0.50, 1.39]	1.15	[0.77, 1.71]	0.79	[0.53, 1.20]	0.66***	[0.52, 0.83]
Education: middle^c^	1.27	[0.82, 1.95]	1.27	[0.97, 1.67]	0.90	[0.65, 1.26]	1.27**	[1.06, 1.51]
Education: high^c^	1.24	[0.79, 1.95]	0.86	[0.62, 1.21]	0.74	[0.47, 1.18]	1.18	[0.95, 1.44]
In poorest quintile^d^	1.04	[0.70, 1.56]	0.80	[0.58, 1.12]	0.86	[0.62, 1.20]	0.76**	[0.61, 0.96]
Married^e^	1.08	[0.78, 1.50]	1.04	[0.79, 1.37]	1.41	[0.94, 1.97]	1.17	[0.98, 1.39]
SRH as poor or fair^f^	0.85	[0.57, 1.29]	0.92	[0.68, 1.25]	0.77**	[0.58, 1.01]	0.74***	[0.60, 0.91]
ADL limitations^g^	1.00	[0.52, 1.94]	0.83	[0.50, 1.36]	1.26	[0.73, 2.16]	0.95	[0.76, 1.21]
Informally engaged at Wave 1^h^	3.41***	[2.56, 4.54]	4.50***	[3.55, 5.70]	5.14***	[4.01, 6.60]	4.47***	[3.87, 5.16]
Formally engaged at both waves	1.00	—	1.00	—	1.00	—	1.00	—
Stopped formal by Wave 2	0.55**	[0.32, 0.94]	0.65**	[0.43, 0.98]	0.73	[0.44, 1.16]	0.62***	[0.45, 0.84]
Started formal by Wave 2	1.04	[0.69, 1.57]	0.88	[0.56, 1.36]	0.61	[0.34, 1.10]	1.02	[0.77, 1.35]
No formal at either wave	0.62***	[0.43, 0.88]	0.64***	[0.46, 0.88]	0.54***	[0.34, 0.85]	0.55***	[0.44, 0.68]
In paid work at both waves	1.00	—	1.00	—	1.00	—	1.00	—
Stopped paid work by Wave 2	1.19	[0.26, 1.29]	1.10	[0.37, 1.17]	1.54**	[1.08, 2.42]	1.38**	[1.06, 1.79]
No paid work at either wave	1.39	[0.69, 1.53]	0.97	[0.70, 1.33]	1.30	[0.86, 1.68]	1.37***	[1.13, 1.65]
Constant	0.30***	[0.16, 0.58]	0.26***	[0.16, 0.44]	0.29***	[0.15, 0.55]	0.11***	[0.07, 0.16]
No. of observations	1,086		1,916		1,758		7,521	

*Source*. Survey of Health, Aging, and Retirement in Europe (2004, 2006); English Longitudinal Study of Aging (2002, 2004). Estimates are based on 20 imputed data sets under missing at random.

*Note*. Study samples are restricted to those who responded to both interviews. Measures of engagement relate to past week in Denmark, France, and Italy and past month in England. Own calculations. Odds ratios (*OR*s) and 95% confidence intervals (CIs) obtained from fully adjusted logistic regression, by country.

^a^Male. ^b^50–54. ^c^Low education. ^d^In other wealth quintiles at baseline. ^e^Not married at baseline. ^f^Self-rated health (SRH) at baseline = good, very good, or excellent. ^g^No activities of daily living (ADL) limitations at baseline. ^h^Not formally engaged at baseline.

** and ***Significant at the .05 and .01 levels, respectively.

Overall, the results of both cross-sectional and longitudinal analysis indicate that formal and informal engagement are positively associated, suggesting complementarity between these two forms of engagement. With regard to paid work, our results suggest that whereas in Denmark and France respondents who stopped working between waves were more likely to have taken on formal activities, in Italy and England they were more likely to have started providing care and help. This suggests contextual influences on activity patterns after retirement. In all countries, however, participation in either formal or informal activities at Wave 2 was strongly predicted by engagement at baseline.

## Discussion

This article aimed to investigate cross-sectional and longitudinal associations between older people’s engagement in three types of activity (paid work, formal, and informal activities) in four European countries. In particular, given the recent emphasis on extending working lives among older people and the mixed evidence on how retirement and work interact with engagement in other spheres, we investigated among individuals aged 50–69 whether paid work was associated with lower odds of being engaged in other activities and whether older people were more or less likely to engage in formal or informal activities following withdrawal from paid employment. A further issue examined was whether associations between activities were similar in four European countries with different institutional and sociopolitical contexts.

Cross-sectional analysis showed that levels of engagement in paid work, formal, and informal activities varied substantially across the four countries under study: for instance, the proportion of Danish respondents engaged in formal activities was 3 times than that of respondents in Italy. Independent of the general level of participation in a country, however, both cross-sectional and longitudinal analyses showed that the association between informal and formal engagement was positive, suggesting complementarity between these two forms of engagement. Participation in informal activities may provide a bridge to other forms of activity, for instance, increasing contacts with social networks and organizations which provide information about and opportunities for formal engagement such as volunteering; it is also possible that people motivated to take part in formal activities may be more likely to also be involved in helping family, friends, and neighbors (Burr, Choi, Mutchler, & Caro, 2005; Burr, Mutchler, & Caro, 2007; Caro, Bruner-Canhoto, Burr, & Mutchler, 2005; Choi, Burr, Mutchler, & Caro, 2007; Hank & Stuck, 2008; Kohli et al., 2009).

Being in paid work was, however, negatively associated with engagement in formal and informal activities, although some differences between full-time and part-time workers were found. Although some studies have reported that part-time work is associated with higher participation in activities such as volunteering (Mutchler et al., 2003; Vickerstaff, 2010); in our study, we found that Danish, French, and English respondents in paid work were less likely to be actively engaged in formal activities regardless of whether they worked part time or full time. Associations between being in paid work and engagement in informal activities were also negative, although in Denmark, France, and Italy only significantly so for those in full-time work. Although this weaker association between part-time work and lower engagement in informal activities might suggest that part-time employment could help those who provide informal care to maintain their employment and combine both activities, as suggested in some other studies (Da Roit & Naldini, 2010; Gordon & Rouse, 2013), care should be taken not to overinterpret this negative finding given the small numbers of part-time workers included in our analyses. Being no longer in paid work increased the likelihood of involvement in other activities in all the countries considered, suggesting that involvement in paid work constrains participation in formal and informal activities. The effect of changes in work status on the other two forms of engagement differed by country. Those no longer in paid work were more likely to become engaged in formal activities in France and Denmark and in informal activities in Italy and England. Such differential associations as well as differences in levels of engagement may be related to different institutional, sociopolitical, and cultural contexts (Hank, 2010; Hank & Stuck, 2008; Pichler & Wallace, 2007; Salamon & Sokolowski, 2001). For instance, the fact that older Italian respondents who stop being in paid work are more likely to engage in informal activities may reflect the important role older people play as providers of family care, and the fact that measures to encourage employees or retired older people to volunteer are less prevalent than in many other European Union countries (Ehlers, Naegele, & Reichert, 2011). In Denmark, where a culture of association is more established and where the government provides a national fund (Satspuljen) to promote active aging and support voluntary initiatives and programs for older people, older people are more likely to start engaging in formal activities (Ehlers et al., 2011; Warburton & Jeppsson Grassman, 2011). However, the results for England, compared with SHARE countries, may also reflect the differences in measures of both formal and informal activities used.

The effect of being no longer in paid work on engagement in other activities, however, was slight compared to the importance of individuals’ previous participation. Baseline participation in formal and informal activities was the strongest predictor of engagement at follow-up, suggesting that older people who are active are likely to stay active. For example, among baseline respondents with no formal engagement (three quarters of the overall sample), only about 15% had taken up participation in formal activities by the second wave. Among those formally engaged at baseline, however, roughly 60% were still involved in formal activities 2 years later. Thus, even following exit from the labor market, most individuals maintain preretirement types of participation. These findings are consistent with other studies suggesting that formal or informal engagement are not simply substitutes for paid employment in later life and that past experiences have a strong effect on the likelihood of engagement in later life (Erlinghagen, 2010; Hank & Erlinghagen, 2010; Kohli et al., 2009; Mutchler et al., 2003). Results therefore lend support to continuity theory (Atchley, 1989), which posits that older people maintain their social roles and social activities throughout their lives.

### Strengths and Limitations

Contributions of the study include the use of data from four samples of older people in Denmark, France, Italy, and England in order to assess associations across socioeconomically and geographically diverse populations and gain a more robust understanding of these associations, while accounting for potentially different confounding patterns. The relationship between paid work, formal, and informal engagement may vary across countries, and findings obtained from pooled data may not reflect the associations existing in each country under study (Allerbeck, 1977). Moreover, whereas previous studies have mostly focused on caring for a sick person and volunteering because these activities were the ones most prevalent at older ages, in this article, we included any continuing participation in social, cultural, economic, and civic affairs, embracing the idea that older people make a contribution to society by maintaining an active role in a wider range of activities, with no underlying assumption of “hierarchy of activities” (Walker & Maltby, 2012).

This analysis, however, has some limitations. The measurements considered in this study were sensitive to the time frame they referred to (i.e., month or week prior to and day of interview). Although highly comparable in many regards, the SHARE and ELSA questionnaires did not collect the same information on formal and informal activities; some of the observed differences in the prevalence of and associations between types of engagement in England might therefore be due to data collection differences. Moreover, data rely on self-reports: measurements such as SRH or participation in certain activities may be sensitive to cultural differences of definitions, and cross-country comparisons should be made with caution (Jylhä, Guralnik, Ferrucci, Jokela, & Heikkinen, 1998).

The study used summary indicators of the various forms of engagement and did not investigate possible effects of concurrent participation in various combinations of activities. Moreover, in Wave 2 of SHARE, the category “provided help to family, friends, and neighbors” dropped the word “family”: This change in questionnaire needs to be taken into account when interpreting the results on the dynamics of informal care described above. In addition, it was not possible to consider all possible work transitions between baseline and follow-up. In particular, we were not able to assess whether returning to work was associated with decreased odds of formal or informal engagement; whether reductions in number of hours worked are associated with increases in engagement in other activities; and whether patterns would be similar for different types of activities and in different countries. Also, the category “not in paid work” is very broad: Although retired people represent about two thirds of the nonworking sample in the age-group under study, this category also included those who described themselves as homemakers, long-term sick, or unemployed. Patterns and levels of engagement may vary considerably between these groups. However, given the striking differences between countries in the number and percentage of respondents who classified themselves as sick, homemakers, or unemployed (for instance, only 18 respondents were “homemakers” in Denmark compared to almost a third of nonworkers in Italy), it was not possible to investigate participation patterns for these subgroups. Sensitivity analyses in which we excluded those who had never worked showed similar findings.

Results from our longitudinal analysis indicate that ending paid work was associated with increased uptake of formal or informal engagement. However, the causal relationship between stopping paid work and engaging in other activities is difficult to disentangle, even in longitudinal studies. For instance, respondents might have started informal engagement after they withdrew from the workplace or they might have withdrawn from paid work because they started caring for a family member.

Both SHARE and ELSA have suffered from attrition. In this study, we used MIs under the assumption that all variables responsible for missingness are included in the estimation. This MAR assumption is reasonable, given that we included in the analysis a wide range of indicators of health and socioeconomic status which are known to be predictive of study drop out, but even so there may be omitted factors associated with attrition leading to some bias.

Finally, none of the data sources provided enough information to capture the *full* complexity of the experiences and activities older people engage in. For instance, we were not able to consider reported benefits and satisfaction derived from activities, as information on this was not available for all the data sets. As former involvement in formal and informal activities fosters the continuing commitment, further work is needed to identify the factors which draw individuals into such activities and motivated them to continue their engagement, as well as barriers that influenced their withdrawal from formal or informal engagement. Although it seems clear that engagement is embedded in the social and economic system of each country, additional research is required to establish to what extent specific programs and strategies, social expectations, financial, or personal rewards are likely to explain country differences in the level of engagement.

## Conclusion

Results seem to suggest that paid work is unlikely to be combined with other activities such as active participation in educational programs, volunteering, and informal care. The current lengthening of working life might therefore raise concerns about older peoples’ reduced time and opportunity to engage in activities other than paid work, calling for an urgent need to identify and foster new ways in which balances between work and other forms of engagement can be achieved for those in early old age. At the same time, in line with earlier studies, this article points to the importance of continuity: The engagement of older people in formal and informal activities is mostly a result of previous experience. Fostering activity in early old age well before retirement might have positive effects on activity later in the life course, although this is a topic that needs further investigation.
